# The Kynurenine Pathway—New Linkage between Innate and Adaptive Immunity in Autoimmune Endocrinopathies

**DOI:** 10.3390/ijms22189879

**Published:** 2021-09-13

**Authors:** Anna Krupa, Irina Kowalska

**Affiliations:** Department of Internal Medicine and Metabolic Diseases, Medical University of Bialystok, M. Sklodowskiej-Curie 24A, 15-276 Bialystok, Poland

**Keywords:** indoleamine 2,3-dioxygenase 1 (IDO1), kynurenine pathway (KP), innate immunity, adaptive immunity, autoimmune disease, autoimmune endocrinopathies, type 1 diabetes mellitus (T1DM), autoimmune thyroiditis

## Abstract

The kynurenine pathway (KP) is highly regulated in the immune system, where it promotes immunosuppression in response to infection or inflammation. Indoleamine 2,3-dioxygenase 1 (IDO1), the main enzyme of KP, has a broad spectrum of activity on immune cells regulation, controlling the balance between stimulation and suppression of the immune system at sites of local inflammation, relevant to a wide range of autoimmune and inflammatory diseases. Various autoimmune diseases, among them endocrinopathies, have been identified to date, but despite significant progress in their diagnosis and treatment, they are still associated with significant complications, morbidity, and mortality. The precise cellular and molecular mechanisms leading to the onset and development of autoimmune disease remain poorly clarified so far. In breaking of tolerance, the cells of the innate immunity provide a decisive microenvironment that regulates immune cells’ differentiation, leading to activation of adaptive immunity. The current review provided a comprehensive presentation of the known role of IDO1 and KP activation in the regulation of the innate and adaptive arms of the immune system. Significant attention has been paid to the immunoregulatory role of IDO1 in the most prevalent, organ-specific autoimmune endocrinopathies—type 1 diabetes mellitus (T1DM) and autoimmune thyroiditis.

## 1. Introduction

Epidemiological studies show that 3–5% of the general population suffers from autoimmune diseases, increasing every year. The pathophysiology of autoimmune diseases usually results from the loss of self-tolerance, leading to the production of autoantibodies and self-reactive lymphocytes that cause tissue destruction. Until now, about 80 distinct autoimmune diseases have been described—several of them are characterized by organ-specific immune dysfunction (such as Hashimoto’s disease (HD), type 1 diabetes mellitus (T1DM)), while the others are systemic immune dysfunction involving multiple organs, like systemic lupus erythematosus, multiple sclerosis, and others [1,2].

Almost half of the diagnosed autoimmune diseases are autoimmune endocrinopathies, of which the most common are thyroid diseases, T1DM, celiac disease, and vitiligo. The consequence of the autoimmune process is, typically, endocrine gland insufficiency; however, the only known exception is Graves’ disease (GD), in which the thyroid gland is not destroyed, yet becomes overactive due to the presence of specific antibodies. Autoimmune endocrinopathies could coexist in the same individuals. Furthermore, its familial occurrence is often observed. Pathophysiology results from a complex interplay among genetic predisposition and environmental/endogenous factors. The measurement of organ-specific autoantibodies and appropriate hormone assessment plays a crucial role in the diagnostic process and treatment strategy [3].

HD is an autoimmune thyroiditis, characterized by thyroid follicular cell atrophy, lymphocytic infiltration within the inflamed organ, and progressive fibrosis [4]. The initial stage of HD may be asymptomatic, while some patients would only have anti-thyroglobulin antibodies (anti-Tg). The appearance of anti-thyroid peroxidase antibodies (anti-TPO) is considered as a predictive factor that indicates the transition of subclinical hypothyroidism into overt hypothyroidism, observed in approximately 20–30% of patients with autoimmune thyroiditis [5].

GD is the most frequent cause of hyperthyroidism in iodine sufficient areas. Production of autoantibodies against the TSH-receptor (TRAb) represents the evidence for disease progression; however, the factors determining the induction of disease remain unknown so far [6]. GD affects the functioning of the majority systems in the human body and usually leads to the development of a clinical symptoms of hyperthyroidism, vascular goiter, Graves orbitopathy (25% of cases), thyroid dermatopathy (about 4% cases); therefore the signs and symptoms associated with GD can vary strongly, and significantly influence the general well-being [7,8].

T1DM is characterized by aberrant immune responses to specific β-cell autoantigens, resulting in insulin deficiency and hyperglycemia, which develops through the interplay of genetic susceptibilities and environmental factors. Although the etiology of T1DM is not completely understood, the pathogenesis of the disease is thought to involve the autoimmunological destruction of β-cells [9]. The peak incidence of T1DM diagnosis is seen in childhood and adolescence [10], nevertheless, symptoms could develop throughout the lifespan. About 90% of cases of newly diagnosed T1DM have detectable antibodies against specific β-cell proteins, like insulin, insulinoma antigen 2, glutamate decarboxylase, tetraspanin-7, or zinc transporter 8 [11]. However, most people with a single autoantibody do not progress to T1DM. The presence of two or more serum autoantibodies in children is associated with an 84% risk of clinical T1DM by the age of 18 years [12]. Based on these observations, the pathogenesis of T1DM was divided into three stages: stage 1 (presymptomatic) is defined as the presence of two or more autoantibodies with normoglycemia, stage 2 (presymptomatic) as the presence of β-cell autoimmunity with abnormal glycemia, and stage 3 as the onset of symptomatic disease [13]. The indicated T1DM pathogenic stages allows for the predictability of disease progression in at-risk individuals and provides a framework for research and development of preventive therapies.

Certain autoimmune diseases occurring in parallel can form into specific syndromes called autoimmune polyendocrine syndrome (APS), which could be defined as a functional disorder of two or more glands. APS type 1 is characterized by Addison’s disease coexisting with mucocutaneous candidiasis, and autoimmune hypoparathyroidism; however, it can also present with T1DM, GD, hypogonadism, vitiligo, or pernicious anemia. APS type 2 can present with Addison’s disease, autoimmune thyroiditis, T1DM, hypogonadism, vitiligo, myasthenia gravis, and alopecia. APS type 3A is associated with T1DM and autoimmune thyroiditis, nevertheless also with growth hormone deficiency and other abnormalities, whereas, in APS type 3C, T1DM is associated with psoriasis and celiac disease [14,15,16].

Autoimmune Addison’s disease (AAD) is known as a dominant component of APS1 and APS2. Furthermore, AAD is the major cause of primary adrenal insufficiency, which is diagnosed with low basal serum cortisol, high plasma adrenocorticotropic hormone (ACTH) concentrations, and impaired cortisol secretion after ACTH stimulation test. Another essential condition for the diagnosis is the presence of autoantibodies to 21-hydroxylase (21-OHAbs); however, adrenal cortex autoantibodies may also be detected in 40–80% of patients with ADD. Due to the destructive autoimmune process resulting in a complete deficiency of cortisol secretion, AAD patients require lifelong hydrocortisone replacement therapy [17].

All of the autoimmune diseases share common pathogenesis, which contains an immune-mediated attack which leads to the destruction of the body’s own organs. It should be mentioned that the innate and adaptive immune system is involved in this process, which can be confirmed in immunological, genetic, and histopathological studies [18,19,20,21,22,23,24]. The kynurenine pathway (KP) of tryptophan metabolism is an endogenous system with immunosuppressive features, which is involved in the control of inflammation and inducing long-term immune tolerance in the different organs across the body [25,26,27]. In this review, we focus on the contribution of indoleamine 2,3-dioxygenase-1 (IDO1) and tryptophan’s catabolites—kynurenines—to regulate the interactions between components of the innate and adaptive immune system. Special attention was paid to the role played by IDO1 and KP metabolites in the onset and progression of autoimmune endocrinopathies.

## 2. The Kynurenine Pathway

In the last two decades, a theory has emerged that metabolism of TRP via KP is involved in the control of immune responses, to keep autoimmunity in check [28,29,30]. TRP is an essential amino acid critical for protein synthesis and the generation of several bioactive compounds with important physiological functions, including serotonin, tryptamine, indoles, kynurenines, and nicotinamide adenine dinucleotide (NAD+) [31]. Humans lack the biochemical pathways to synthesize TRP, which must be gathered from the diet. After absorption of TRP via enterocytes in the gut, it is transported by the hepatic portal system into the liver, where is utilized for protein synthesis (less than 1% of ingested TRP), whereas about 95% of dietary-delivered TRP is metabolized via the KP in the liver. The remaining TRP is secreted into the bloodstream and is available to use by cells of peripheral tissues, such as the vascular endothelial cells, fibroblasts, and the cells of innate immunity [32]. Moreover, TRP can also be transported across the blood–brain barrier to regulate brain serotonin synthesis [33].

**Figure 1:**
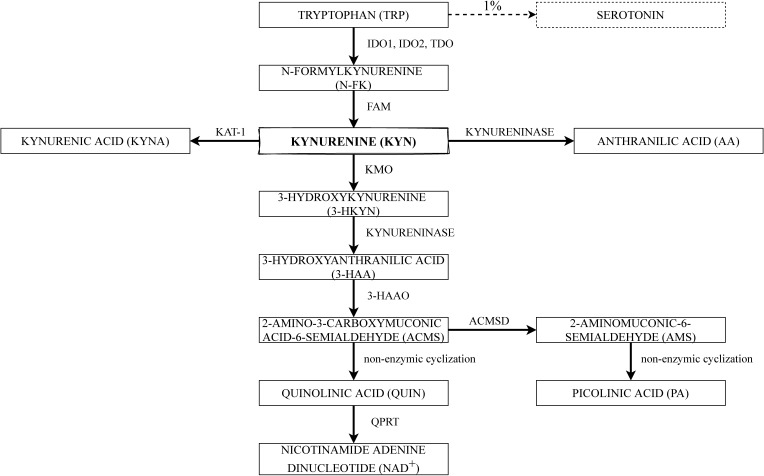
Schematic representation of the kynurenine metabolic pathway. Abbreviations: FAM—N-formylkynurenine formamidase; IDO1—indoleamine 2,3-dioxygenase 1; IDO2—indoleamine 2,3-dioxygenase 2; TDO—tryptophan 2,3-dioxygenase; KMO—kynurenine 3-monooxygenase; 3-HAAO—3-hydroxyanthranilate 3,4-dioxygenase; ACMSD—2-aminomuconic acid semialdehyde dehydrogenase; QPRT—quinolinic acid phosphoribosyltransferase.

The kynurenine pathway is the main way of TRP metabolism [34]. The major enzymes and substrates of the KP are shown schematically in Figure 1. To begin with, TRP has to be converted into N-formylkynurenine, which is mediated by indoleamine 2,3-dioxygenase (IDO) and tryptophan 2,3-dioxygenase (TDO), and then into kynurenine (KYN) by N-formylkynurenine formamidase (FAM) [35]. The first step in TRP degradation under normal conditions is mediated by TDO, which is the main determinant of TRP extrahepatic availability and is inducible by TRP itself, estrogens, and glucocorticoids. However, under a high cortisol concentrations and inflammatory state, TDO expression in the liver is repressed, whereas IDO1 expression is induced in cells of the immune system, as part of a negative feedback loop, aiming to control inflammatory responses [36].

The extrahepatic KP remains under the control of two distinct IDO enzymes: IDO1 and IDO2, the activities of which may differ from each other. The activity of IDO1 is irrelevant under basal conditions, but strongly inducible by several inflammatory stimuli, such as interferon-γ (IFN-γ), lipopolysaccharide (LPS), tumor necrosis factor α (TNF- α), proinflammatory interleukins (ILs), infection, and transforming growth factor β (TGF-β) [37,38]. IDO1 is mostly active in the immune system cells, mucosal tissues, and some tumors; however, it could be inhibited by elevated TRP levels. The anti-inflammatory cytokines, IL-4 and IL-13, are causing a down-regulation of IDO1 mRNA expression and reduction of TRP catabolism [39], although controversial data concerning the role of IL-4 also have been reported [40]. The enzymatic activity of IDO2 is approximately 500–1000-fold lower than that of mammalian IDO1, and IDO2 is mainly expressed in the liver, epididymis, and kidney [41]. Current studies showed a multifarious and pivotal role of IDO1 in immunoregulation during infection, pregnancy, autoimmune diseases, and neoplasia of various origins [26,28,42,43].

TDO is considered as a “higher catalytic activity” enzyme in comparison to IDO1 [32]; however, IDO1 has broader substrate specificity than TDO. The main sources of TDO in the human body are the liver and central nervous system [44], nevertheless, it has also been identified in mucous membranes, epididymis, and the brain [45].

KYN and its metabolites are biologically active. Consequently, their production must be strictly controlled. KYN is the central intermediate of the KP, where the metabolic pathway is divided into two different branches. KYN may be converted by kynurenine 3-monooxygenase (KMO) into 3-hydroxykynurenine (3-HKYN), which is known as one of the toxic metabolites. Human KMO is a protein, which requires nicotinamide adenine dinucleotide phosphate (NADPH) for its catalytic action [46]. Ensuing, kynureninase can convert 3-HKYN into 3-hydroxyanthranilic acid (3-HAA). Nevertheless, kynureninase could as well convert kynurenine directly into anthranilic acid (AA) [47]. In general, the last step of the KP is the conversion of 3-HAA into quinolinic acid (QUIN) by 3-hydroxyanthranilate 3,4-dioxygenase (3-HAAO) through the unstable product of this reaction—2-amino-3-carboxymuconate-6-semialdehyde (ACMS)—which further undergoes a nonenzymic cyclization to QUIN. Picolinic acid (PA) is also formed by a nonenzymic cyclization of aminomuconic acid semialdehyde (AMS). However, PA formation depends on the extent of the substrate saturation of the enzyme 2-aminomuconic acid semialdehyde dehydrogenase (ACMSD) [35]. Finally, QUIN is processed into end product NAD+ by quinolinic acid phosphoribosyltransferase (QPRT) [48].

Nonetheless, another branch of KP is also known—it is minor under regular conditions, whereas increases while TRP or KYN profusion, and contains a transformation of KYN into kynurenic acid (KYNA), which is also recognized as an endogenous antagonist of *N*-methyl-d-aspartate (NMDA) receptors. The above-mentioned step is catalyzed by kynurenine aminotransferase 1 (KAT-1) [48,49].

## 3. The Role of IDO1 and KP Metabolites in Immune System Regulation

### 3.1. The Innate and Adaptive Immunity

The immune system continuously maintains the sophisticated balance between invading pathogens and tolerance to non-harmful antigens and self-antigens. As an entirety, the immune system consists of innate and adaptive immunity, each responsible for a different capacity and constitutes diverse cellular and non-cellular components [50]. Innate immunity is the first line of defense and provides the initial acute inflammatory reaction to tissue injury, foreign antigens or pathogens [51]. Innate immunity is to a certain extent unspecific and is divided into cellular and non-cellular systems. The cellular components of the innate system include monocytes/macrophages, dendritic cells (DCs), natural killer (NK) cells, eosinophils, and neutrophils. The non-cellular system is extremely diverse—it recruits immune cells to the injury/infection site through various cytokines, promotes phagocytosis, and activates the complement cascade and adaptive immune system [51,52].

The activation of the adaptive immune system results in an antigen-specific host response that is mediated by T and B cells. B cells secrete antigen-specific antibodies to neutralize pathogens, mediate allergic reactions, autoimmunity and generate immune memory cells. T cells are involved in the production of cytokines, direct cytotoxic effect against infected tissue, and activation of the other immune cells [50]. Cellular cross-talk is a hallmark of the adaptive immunity. The proliferation and differentiation of naive B cells in response to most antigens must be preceded by stimulation via T cells, that are specific for the same antigens. Similarly, T cells in order to proliferate in response to antigens need additional signals provided by B cells [50]. Thus, innate and adaptive immunity work together to establish and maintain tissue homeostasis. Any sort of dysregulation could disturb regular immune response and result in persistence of chronic inflammation, or even induce autoimmune responses in more susceptible individuals.

### 3.2. Kynurenines in the Immunoregulation—“TRP Depletion Theory” versus “TRP Utilization Theory”

Recently, the role of KP in the regulation of both innate and adaptive immune responses does not raise any doubts, although it is still not fully explained. In the past, two opposing theories persisted, referring to the importance of TRP metabolism via KP in immunoregulation. The first, “depletion theory” assumed that TRP depletion is the primary function of immune-related IDO1 induction, which has been recognized as a host defense mechanism of innate immune responses. Pfefferkorn showed that the growth of *Toxoplasma gondii* could be inhibited by IFN-γ-mediated IDO1 induction, which was associated with decreasing TRP concentrations [53]. In the other in vitro studies, the replenish of TRP concentrations to the culture media restored the growth of cancer cells, bacteria, and parasites, supporting the TRP depletion theory [54]. This theory transformed while Munn et al. [55] discovered that IDO1 activity was required to prevent T cell mediated rejection of allogenic fetuses in pregnant mice. They also found that T cells proliferation may be inhibited in vitro by stimulation of cocultured monocytes with IFN-γ, which induced IDO1-mediated depletion of TRP from the culture medium. The later study of Lee et al. [56] demonstrated that T cells, activated in the absence of TRP, entered the cell cycle; however, cell cycle progression is arrested in the G1 phase and T cells became susceptible to death via apoptosis, in part through Fas-mediated signaling. Moreover, reduced availability of TRP has been correlated with activation of the general control nondepressible 2 kinase (GCN2K) pathway, inhibition of the mammalian target of rapamycin (mTOR), and protein kinase C signaling, leading to T cell autophagy and anergy [57]. According to the present conceptualization, TRP depletion acts to limit the proliferation of specific host cells, that became more susceptible to apoptotic stimuli [56].

TRP depletion hypothesis only explained IDO1 activation, whereas during an immune response both KYN, as well others, downstream KYN metabolites: 3-HKYN, 3-HAA, PA KYNA, and QUIN are generated in many tissues [43]. These metabolites were shown to be potent in the inhibition of T cell proliferation through induction of T cell apoptosis. The study using a heart transplantation model in rats confirmed these results in vivo [58], forming the basis for the so-called “TRP utilization theory” [59]. The indicated theory assumed that the immunomodulatory properties of IDO1 are due to the accumulation of KYN metabolites in conjunction with TRP depletion [32].

### 3.3. Immunoregulatory Activity of IDO1

**Figure 2:**
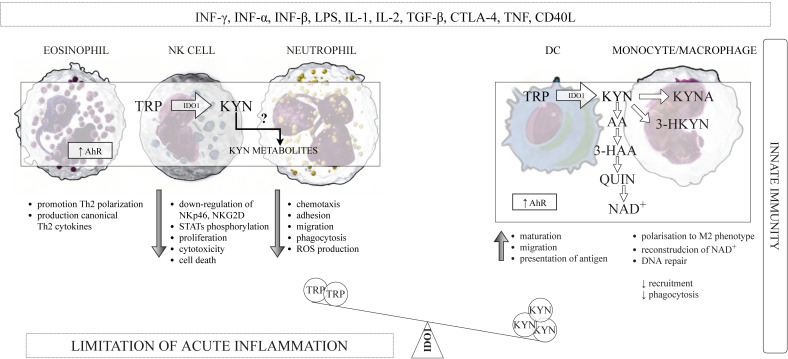
The role of the indoleamine 2,3-dioxygenase 1 (IDO1) and kynurenine pathway metabolites in innate immunity. In acute inflammation, IDO1 is expressed in cells of the innate immunity under the influence of various pro-inflammatory factors. The active IDO1 enzyme transforms local TRP to KYN, and potentially could be transformed into other KP metabolites, depending on the cell type and its expression of downstream enzymes of the KP. Antigen-presenting cells (DC, monocyte, macrophage) are equipped in all enzymes of KP, and especially in these cells, KYN may be further metabolized. Kynurenines derived from IDO1-mediated TRP degradation can activate AhR, which is present in all cells belonging to the innate immunity. Induction of IDO1 expression and activity, as well as the metabolites of KP, have multiple effects on innate immunity. DCs maturation, migration, and antigen presentation are dependent on IDO1 activity. However, IDO1 plays a suppressive role in the immune responses generated by monocyte/macrophages, eosinophils, neutrophils, and NK cells, contributing to the limitation of the local inflammatory state. Abbreviations: IFN-α—interferon α; IFN-β—interferon β; IFN-γ—interferon γ; LPS –lipopolysaccharide; IL-1—interleukin 1; IL-2—interleukin 2; TGF-β—transforming growth factor β; CTLA-4—cytotoxic T-lymphocyte antigen 4; TNF—tumor necrosis factor; CD40L—CD40 ligand; NK—natural killer; DC—dendritic cell; TRP—tryptophan; KYN—kynurenine; IDO1—indoleamine 2,3-dioxygenase 1; AA—anthranilic acid; 3-HAA—3-hydroxyanthranilic acid; QUIN—quinolinic acid; NAD^+^—nicotinamide adenine dinucleotide; KYNA—kynurenic acid; 3-HKYN—3-hydroxykynurenine; AhR—aryl hydrocarbon receptor; Th2—T helper type 2 cell; M2—macrophage type 2; NKp46—natural toxic receptor; NKG2D—C type lectin receptor; ROS—reactive oxygen species; STATs—signal transducers and activator of transcription.

IDO1 is widely expressed in a variety of cells that belong to the immune system, such as macrophages, monocytes, DCs, eosinophils, neutrophils, some T cells subsets, and regulatory B cells [60,61,62,63,64,65]. The induction of IDO1 expression and activity in professional antigen-presenting cells (APCs), such as DCs and monocyte-derived macrophages, as well as in other components of the innate immune system—NK cells, eosinophils, and neutrophils have a multidirectional influence on the function of these cells in the immune system (Figure 2).

#### 3.3.1. IDO1 and DCs

Dendritic cells are professional APCs and key regulators of the immune system. DCs perform many functions in the immune system, including uptake, processing, and presentation of antigens to naive T cells, the activation of effector T cells and NK cells, and secretion of cytokines and other immune-modulating molecules to shape T and B cell responses. Two major subsets of human peripheral blood DCs have been described: conventional DCs (cDCs) and plasmacytoid DCs (pDCs) [66]. pDCs represent a unique cell population, combining the innate and adaptive immune responses in defense against pathogens, autoimmunity and cancer [67]. pDCs secrete large amounts of type I and III interferons and are able to secrete IL-6, IL-12, IL-23, TNF-α, and interferon-inducible protein 10 (IP-10). They also express major histocompatibility complex class II (MHC-II), MHC-I, and co-stimulatory molecules (CD40, CD80, CD86) for antigens presentation [67,68]. The production above mentioned molecules allows pDCs to shape the type of immune response. For example, IL-12 can induce Th1 response and CD8^+^ T-cell and NK-cell activation, which are important for combating viral and intracellular pathogens’ infection, whereas IL-6 and IL-23 may direct the immune activity towards a Th17 response, which plays an important role in the recruitment of neutrophils and macrophages, immune responses against fungal infections and in autoimmune diseases [69]. pDCs may also exert direct effector functions. They may express the TNF-related apoptosis-inducing ligand (TRAIL), which causes TRAIL-sensitive cell death [70]. Moreover, pDCs can kill target cells by releasing the serine protease granzyme B [71]. Recently, the role of pDCs in autoimmune disease has been proposed. pDCs may act directly on the differentiation/maintenance of autoreactive B cells, and promote autoreactivity indirectly through T cells or other cell types [72]. On the other hand, an impaired pDC activity has been implicated in immunodeficient states or ineffective immune responses [73].

Although DCs play an essential role in the initiation of inflammatory responses, they are also able to induce immunotolerance, inter alia through the upregulation of the intracellular enzyme IDO1. These cells express both constitutive and IFN-γ-inducible forms of the enzyme [74,75]. In particular, pDC have been shown as having the ability to produce a high amount of IDO1 [60]. Despite this, pDCs have been described as rather poor at their antigen-presenting function in comparison to cDCs [76]. IFN-γ alone can induce up-regulation of IDO1 message in DCs; however, an additional stimulus, such as CD40L or LPS, results in significantly higher IDO1 expression [75]. Aryl hydrocarbon receptor (AhR) activation in DCs is the following important factor for IDO1 expression in these cells. KYN and other KP metabolites—3-HKYN and KYNA—are found to be endogenous ligands for the AhR and this mechanism may determine a tolerogenic DCs phenotype, which promotes Tregs expansion [77,78]. It seems that IDO1 expression in pDCs may rather modulate the immune response of effector cells, as depletion of IDO1-expressing pDCs resulted in increased T cell proliferation and intensification of inflammation [79]. The aforementioned finding was confirmed in numerous studies, in which IDO1- expressing DCs function as part of a “feedback” process to limit chronic or over-activation of the immune system. DCs producing IDO1 can suppress effector T cells proliferation and may induce T cells apoptosis [75,80]. IDO1-expressing pDCs mediate the down-regulation of the receptor zeta-chain in T cells and promote the expansion of forkhead box P3^+^ (Foxp3^+^) T regulatory cells (Tregs) [81]. Expression of IDO1 in DCs can also skew CD4^+^ T-helper cells from proinflammatory phenotype Th1 or Th17 to tolerogenic Tregs [82]. Thus, IDO1 expression by DCs is associated with peripheral tolerance and the induction of immunosuppression.

Several molecules that induce immune suppression/tolerance have been shown to mediate their activity via IDO1. The ligation of B7 molecules on the DCs with the cytotoxic T-lymphocyte- antigen 4 (CTLA-4), a co-inhibitory molecule expressed on Tregs, can induce IDO1 expression in DCs [83,84]. Interactions between programmed death 1 (PD-1) receptors on T cells with its ligands on the DCs can also promote the up-regulation of IDO1 [85]. Additionally, the immunosuppressive TGF-β could elicit and maintain IDO1 expression in pDCs [86]. Similarly, the other molecules, like LPS or INF-γ, which are able to induce AhR expression in DCs, can also maintain IDO1 at high levels using this positive mechanism [77,87].

IDO1 possesses the capacity to control DCs maturation, migration, and their immunoregulatory properties. DCs exist in the periphery as immature cells responsible for capturing antigen for priming naive T cells. Upon maturation, DCs migrate to the draining lymphoid organs, where they may initiate immunity. It has been shown that IDO1 expression and activity was increased during DCs maturation, which was related to phenotypic and functional changes essential for generating MHC/peptide complexes and priming T cells [88]. In contrast, IDO1 deficiency led to diminished phenotypic and functional maturation of DCs in vitro and in vivo [89]. However, Bracho-Sanchez et al. [90] showed that DCs treated with exogenous human recombinant IDO maintain an immature phenotype without affecting their viability, and provide suppression of antigen-specific T cell proliferation in vitro. Moreover, IL-12p70 production in DCs was significantly diminished, while IL-10 was maintained, suggesting that naive Th cell differentiation may be directed into immunosuppressive Th2 or Tregs. These results indicate that DCs conditioning was mediated by the enzymatic action of IDO1 and that DC-mediated suppression of T cells was dependent on both TRP deletion and the presence of kynurenines, which together were more effective in the abrogation of T cells stimulation.

#### 3.3.2. IDO1 and Monocytes/Macrophages

Monocytes and macrophages have broad inflammatory, immuno-modulatory, and tissue-repairing properties. They belong to the front line of defense cells and can activate the immune system to trigger an immune response. Prior to polarization, macrophages exist as uncommitted (M0), which will be able to express the specialized functions after the stimulation by appropriate cytokines and microbial products. The stimulation leads to the polarization of M0 cells into 2 groups: M1- and M2-type macrophages, which are recognized as classically and alternatively activated macrophages, respectively. M1 macrophages may be induced by the granulocyte-macrophage colony-stimulating factor (GM-CSF), IFN-γ, and LPS, whereas M2-type macrophages can be polarized after exposition to immune complexes, IL-4, IL-13, IL-10, and glucocorticoids [91]. Typically, M1 macrophages are considered as proinflammatory, and secrete IL-12 and TNF-α, while M2 macrophages possess immunomodulatory, wound repair, and tissue remodeling functions, and produce IL-4 and IL-10 [92]. Macrophages have a high degree of functional plasticity: they can easily switch from M1 to M2—type, and vice versa, depending on the cytokines present in their environment [93]. Nevertheless, in certain autoimmune diseases, both M1 and M2 macrophages, as well as produced by them cytokines were observed simultaneously [92]. Moreover, intermediate forms of macrophages, co-expressing both M1- and M2-specific markers were detected in certain diseases [94]. These findings indicated that macrophage polarization is a dynamic and reversible process that depends not only on the local environment but also on the stage of the disease.

Macrophages and monocytes can express IDO1, but only following IFN-γ stimulation [74]. IDO1 induction is able to switch macrophage phenotype from pro-inflammatory M1 to tolerogenic M2. Wang et al. [61] showed that the expression of IDO1 in M1-type macrophage, differentiated from THP-1 cells treated with IFN-γ, was significantly higher than in M2-type, which polarized from THP-1 cells cultured with M-CSF. They also demonstrated that the overexpression of IDO1 promotes the differentiation of THP-1 cells, widely used as the model for monocytes/macrophages differentiation, to M2-type macrophages. On the contrary, the silence of IDO1 induces the formation of M1-type macrophages [61].

IDO1 can inhibit macrophages recruitment and phagocytosis process in mice model of *Aspergillus fumigatus* keratitis. However, IDO1 may also promote the polarization of macrophages into the M1 phenotype by activating the mitogen-activated protein kinase/extracellular signal-regulated (MAPK/ERK) signaling pathway, indicating that it is essential for keeping the balance between anti- and proinflammatory effects in this model [95]. The diverse role of macrophages in the inflammatory responses may be partly due to the presence of AhR. It has been reported that AhR-deficient macrophages showed a higher level of proinflammatory cytokines upon LPS stimulation and that AhR-deficient mice were more susceptible to LPS-induced lethal shock than wild-type mice [96]. Recently, Suchard et coworkers [97] summarized existing literature and showed that elevated IDO1 activity is regarded as a feature of M2 macrophage activation.

An inflammatory state is characterized by high levels of cellular stress and energy use, which is often accompanied by increased rates of DNA damage. It has been noted that the oxidation of TRP through the KP can reconstruct NAD+ levels to meet energy requirements and support DNA repair mechanisms in macrophages, increasing their viability [98].

#### 3.3.3. IDO1 and NK Cells

NK cells are cytotoxic lymphocytes, which play a significant role in immune responses to exogenous pathogens as well as in the defense against cancer cells. Circulating NK cells mainly appear in the resting phase; however, the stress, as a result of infection or malignancy, causes their activation and the secretion of cytotoxic granules or death receptor ligands [99]. In the activation of NK cells, the activating and inhibitory receptors present on their surface play an important role. The inhibitory receptors consist of the killer immunoglobulin-like receptors (KIR), Ig-like receptors (CD158), the C-type lectin receptors (CD94-NKG2A), and leukocyte inhibitory receptors (LIR1, LAIR-1). Important NK activating receptors include NKG2D, DNAM1, and natural cytotoxic receptors: NKp46, NKp30, NKp44, and CD16 (FcgRIII), which are involved in antibody-dependent cytotoxicity. After binding the appropriate ligands, these activating and inhibitory receptors cooperate and decide whether to exert NK cell cytotoxicity on target cells [100]. The direct cytotoxic effect of NK cells is mainly mediated via two pathways: the induction of apoptosis of target cell by secretion of membrane-disrupting proteins and proteases, or caspase-dependent apoptosis involving the death receptors (e.g., Fas/CD95) on target cells [99].

NK cells, which are one of the main components of the innate immune system, constitute a link between innate and adaptive immunity. Besides their direct cytotoxicity, NK cells release various cytokines and chemokines, such as GM-CSF, IFN-γ, TNF-α, and chemokines: CCL3, CCL4, and CCL5 [101] or crosstalk with other immune cells, like T and B cells and DCs [102,103]. They additionally exhibit immunologic memory which is able to persist upon cognate antigen encounter [104]. The Janus kinase/signal transduction and activator of transcription (JAK-STAT) pathway plays an important role in NK cells’ maturation, cytotoxicity, or survival, and most cytokines that can activate or block NK cells are known to regulate it [105]. It is established that IL-2, which plays an important role in NK cell proliferation and receptor expression, can activate STAT1, 3, and 5. Moreover, STAT5 is activated by IL-15, and that STAT1 and 3 are activated by IL-21, which leads to proliferation, maturation, and activation of NK cells [106]. Therefore, NK cell hyperactivation and dysfunction are associated with the pathogenesis of some inflammatory and autoimmune diseases. However, NK cells could have both protective and pathogenic roles in these diseases depending on the disease type and surrounding environment [107,108].

Kai et al. [109] identified INF-γ—dependent IDO1 mRNA expression in NK cells, and pharmacological inhibition of IDO1 reduced cytotoxicity of NK cells against cancer cells. This finding was confirmed in vivo, in a model of subcutaneous B16 tumors in mice [64]. These results suggested that IDO1 in effector NK cells appeared to maintain the normal cytotoxicity against tumor cells. However, it has been also reported that IDO1 catabolites block the proliferation of NK cells [110]. The recent study of Park et al. [111] showed that activation of IDO1 in tumor cells caused downregulation of the activating natural cytotoxic receptors NKp46 and NKG2D in NK cells, suppressing their cytolytic activity and inducing NK cell death. This destructive effect was mediated by up-regulation of IDO1 and the production of KYN, which enters NK cells via AhR on their surfaces and directly impairs NK cell function. KYN treatment led to the decreased phosphorylation of STAT1 and STAT3 in NK cells in a dose-dependent manner, indicating that KYN regulates NK cells via STATs signaling pathways. In contrast, the pharmacological blocking of IDO1 activity in tumor cells restored NK cells’ cytolytic activity and receptors expression [111]. These data suggest that IDO1 activation in NK cells located in the tumors environment can play an antitumor function, whereas IDO1 produced by tumor cells themselves may act as a negative feedback mechanism against antitumor immune responses.

#### 3.3.4. IDO1 and Eosinophiles

Eosinophils are multifunctional leukocytes that have been implicated in the pathogenesis of the inflammatory processes, including helminth infections and allergic diseases. They have been considered as cells that mainly act as the first-line defense against parasites or can modulate immune responses to diverse stimuli. IL-5, produced primarily by Th2 cells, is a crucial cytokine for eosinophil differentiation, priming, and survival [112]. Nonetheless, eosinophils themselves serve as a source of a variety of cytokines and growth factors closely associated with multiple immuno-modulatory functions, and are involved in numerous homeostatic processes in the thymus, mammary gland, uterus, and gastrointestinal tract [113,114]. They show chemotaxis to lymphoid chemokines and exhibit APCs-like properties upon stimulation with some cytokines. The antigen-presenting properties of these cells are possible thanks to the expression of the machinery for antigen presentation and co-stimulation molecules, including MHC-II, CD80, CD86, CD28, and CD40 [115], as well as with their direct cross-talk with DCs [116]. The ability of eosinophils to antigen presentation and allergen-induced their recruitment to lung tissue has been suggested as evidence of interaction between eosinophils and T lymphocytes [117]. The study of Venge et al. [118] in patients with asthma indicates that eosinophils actively participate in lung tissue fibrosis and remodeling, linking them to the potential etiology of this disease and worsening of quality of patients’ life. On the other hand, it has been shown that eosinophils may participate in tissue repair, as they are equipped with a tissue damage-sensing system, and can release multiple tissue repairing molecules, like different growth factors [112].

Human eosinophils express functionally active IDO1, both constitutively and after IFN-γ induction [62,119], and coculture of KYN- synthesizing eosinophils with IFN-γ-producing T cells, but not IL-4-producing T cell subsets, led to apoptosis and inhibition of Th1 subset proliferation, whereas Th2 cell line was maintained [62]. The same team showed that the pharmacological inhibition of IDO1 in vivo resulted in the reversal of oral immune tolerance in an ovalbumin (OVA)-induced murine model and that repeated intranasal administration of OVA generated tolerance and prevented a subsequent sensitization to OVA [120]. These results indicated that IFN-γ-treated eosinophils can promote Th2 polarization through the expression of functionally active IDO1 in lymphoid tissue. Moreover, eosinophils can be driving a Th2 response by their capacity to produce canonical Th2 cytokines, like IL-4, IL-5, and IL-13 upon stimulation [121]. However, Tulic et al. [122] observed the presence of functional IDO1, which was constitutively expressed in thymic eosinophils during human infant life under non-pathological conditions. Simultaneously, KYN was detected intracellularly and around the cells morphologically resembling eosinophils. The induction of IDO1 and TRP catabolite—KYN—promoted Th2 cells dominance over Th1 cells, which undergo selective apoptosis under these conditions. The above data suggest an immunomodulatory role of IDO1-expressing eosinophils, which may have important implications for adaptive immune development.

#### 3.3.5. IDO1 and Neutrophils

Neutrophils are polymorphonuclear leukocytes and have been shown as one of the essential players during the acute inflammatory states, which can be recruited from the bloodstream to sites of injury within minutes. They eliminate invading pathogens through several mechanisms, such as secretion of bactericidal molecules, engaging in phagocytosis, degranulation, and secretion of proteolytic enzymes and reactive oxygen species (ROS), or release of nuclear material in the form of neutrophil extracellular traps [123]. The circulating neutrophils are typically “resting cells”, and their harmful intracellular granule contents are not released to avoid host tissue injury. However, neutrophils can become primed during immune conditions, where they may exhibit a 10- to 20-fold increase in their response to the proinflammatory stimulation, resulting in aggravate surrounding healthy tissues damage [124]. The excess activation and recruitment of neutrophils have been implicated in the development of various chronic inflammatory conditions, such as rheumatoid arthritis, inflammatory bowel disease, rheumatoid arthritis, metabolic syndrome, atherosclerosis, and cancers [123,125]. On the other hand, neutrophils may also promote wound healing and the limitation of inflammation [126,127].

Apart from the major role of neutrophils in innate immunity, these cells can significantly modulate the main components of adaptive immunity by the impact on B cells and T cells. Neutrophils produce cytokines—the B cell-activating factor (BAFF) and a proliferation-inducing ligand (APRIL)—which are required for the survival and activation of B cells, and stimulation of them to produce antibodies [128]. Neutrophils may produce arginase-1 and ROS, and in this manner, they can inhibit the proliferation and activation of T cells [129]. They can also function as APCs, facilitating Th1 and Th17 differentiation [130], and are able to present antigens directly to T cells or transfer them to DCs [131].

A few neutrophil subtypes have been known, and between them, neutrophilic myeloid-derived suppressor cells (MDSCs) are identified, which play a major role in the regulation of immune responses in cancer and many pathological conditions, associated with chronic inflammation [132]. The precise cellular mechanisms, by which MDSCs can suppress T-cell responses have not been completely explained, but Novitskiy et al. [133] found that the incubation of MDSCs with IL-17 increased the suppressive activity of these cells through the up-regulation of arginase 1, IDO1, and cyclooxygenase-2 expression in mammary carcinoma model in mice. Loughman et al. [134] observed that uropathogenic *Escherichia coli* (UPEC) infection reduced phagocytic killing and dampened the production of antimicrobial ROS by neutrophils, as well as downregulated their proinflammatory signaling, chemotaxis, adhesion, and migration. The same team showed that UPEC attenuated innate responses by inducing IDO1 expression in human uroepithelial cells and neutrophils in vitro, and that treatment of neutrophils with a specific inhibitor of IDO1 significantly enhanced their transepithelial migration in response to UPEC. Moreover, neutrophils function was not affected in IDO1-knockout mice [135]. Similarly, an initial exposure to *Plasmodium vivax* induced activation of innate immunity, but that effect was accompanied by strong immunosuppression mediated by IDO1-expressing DCs, which was associated with depletion of some neutrophil populations. Because neutrophils regulate DCs function during infection, the cross-talk between these cell populations seems to be an important component of the innate immune response [136]. These results indicated that the induction of IDO1 expression in neutrophils inhibits proinflammatory innate responses and promotes pathogen colonization, confirming the role of IDO1 as a critical regulator of early host-pathogen cross-talk. On the other hand, it was also suggested that regulatory Tregs, emerging during IDO1-mediated immunosuppression, were able to promote TGF-β production, as well as IDO1 and heme oxygenase-1 expression by neutrophils. Thus, Tregs may play an important role in the direct control of innate immune responses through the induction of neutrophils with immunosuppressive properties [137].

### 3.4. Kynurenines and the Components of the Innate Immune System

IFN-γ and other Th1 cytokines, such as IL-1, TNF-α, and IL-2, may stimulate the activity of IDO1 [138]. The expression of other enzymes of KP—KMO, KYNU, and 3-HAAO—is also under the control of IFN-γ [139]. Professional APCs, such as DCs, monocytes, and macrophages, are able to express IDO1 following IFN-γ exposure [61,75], and they might also express others enzymes of KP in these conditions. Indeed, it has been shown that all enzymes of the KP are expressed in macrophages [140], and that these cells can produce some kynurenines, including AA, 3-HK, 3-HAA, PA, and QUIN, after activation [141]. The expression of QUIN was observed in peripheral monocytic cells of patients with Alzheimer’s disease [142]. Moreover, the monocyte culture treated with IFN-γ and supplemented with TRP produced KYN and 3-HKYN, and neutrophils produced KYN as well [63]. Similarly, the expression of KP enzymes was demonstrated in human monocyte-derived DCs, which were able to mediate apoptosis of Th cells following stimulation with IFN-γ [143]. McIlroy et al. [144] demonstrated that DCs maturation leads to the formation of KYN, 3-HKYN, and 3-HAA. Taking together, the cells belonging to innate immunity, particularly APCs, can contribute to TRP degradation and accumulation of kynurenines—the TRP-derived metabolites in the vicinity of other cells of the immune system.

It has been shown that KYN metabolites, in particular, KYN itself, suppress the activity of NK cells and APCs. Loughman et al. [145] demonstrated that KYN, 3-HKYN, and 3-HAA impaired neutrophil chemotaxis and directly suppressed their transepithelial migration induced by UPEC. Moreover, TRP catabolism via KP shave a negative impact on cells viability. The accumulation of TRP-derived metabolites is toxic for NK cells and monocyte-derived TPH-1 cells and can induce cells death by apoptosis [110,146]. These effects are, at least in part, mediated by KYN activation of AhR, which is expressed in all cells belonging to the innate immune system.

KYN can induce the production of intracellular IDO1 through the positive feedback loop, for example, KYN can engage AhR in the cytosol of DCs, and KYN-AhR interaction resulted in amplification of IDO1 expression [87], with simultaneous suppression of stimulatory and co-stimulatory molecules expression in DCs, as well as promote the production of anti-inflammatory cytokines by these cells [147]. Similarly, KYNA was also found to activate AhR, but differently from KYN. The interaction KYNA/AhR resulted in the production of proinflammatory IL-6 [148]. However, KYNA is also a ligand for the G protein-coupled receptor 35 (GPR35), which is expressed in human monocytes, neutrophils, DCs, eosinophils, NK cells, and T cells. The interaction KYNA-GPR35 reduces the inflammatory response in monocytes and macrophages induced by stimulation with LPS and controls cytokines release in NK cells [149].

In summary, it appears that IDO1-mediated KP activation in the cells of the innate immunity could beneficially contribute to limit the excessive inflammatory response, protecting local tissue from inflammation-mediated damage.

### 3.5. IDO1, KYN Pathway Metabolites, and the Components of the Adaptive Immune System

#### 3.5.1. T Cells Subsets

T cells are divided into two major types: cytotoxic T cells and T helper cells. T cells expressing the CD4 molecule (CD4^+^T cells) are helper T (Th) cells, whereas T cells expressing the CD8 molecule (CD8^+^T cells) are cytotoxic T cells, which can directly destroy malignant, infected, and senescent cells [150]. Th cells are crucial for immune responses during host defense against detrimental pathogens, but they can also play an important role as drivers of inflammatory and autoimmune diseases [151]. Currently, Th cells can be divided into several subpopulations: Th1, Th2, Th17, Th22, Th9, follicular helper T cells (Tfh), and Tregs, depending on the profile of cytokines they produce [150,151]. The differentiation of each of Th subset depends upon the expression of specific transcription factors: T-bet for the Th1 cells, GATA-binding protein 3 (GATA3) for the Th2 cells, retinoic acid receptor-related orphan receptor-γt (RORγt), AhR for Th17 and Th22 cells, B cell lymphoma-6 (Bcl-6) for Tfh cells, and Foxp3 for Tregs [152]. Th cell subsets are defined by the signature cytokines that they express and their specialized effector functions.

Th1 cells are defined by their production of IL-2 and IFN-γ, but also they produce several cytokines, including TNF-α, lymphotoxin, and GM-CSF. Th1 cells are particularly effective at activating macrophage microbicidal mechanisms against intracellular pathogens. They are involved in cell-mediated inflammation and delayed-type hypersensitivity reactions [150,152].

Th2 cells are the best known for the production of IL-4, IL-5, and IL-13, as well as IL-9 and IL-10. These cells play a role in the elimination of extracellular parasites and involve in allergies and atopic diseases [150,153]. They are mainly responsible for humoral immunity via the activation of B cells, mast cells, and the production of immunoglobulin E. It has been shown that IL-4 expression in vivo can protect autoreactive B cells from apoptosis, enhance their survival, and induce activation of autoreactive B cells [154]. On the other hand, Th2 cytokines can mediate protection against Th1-dependent inflammation or may directly suppress Th1/Th17 development via IL-4/IL-13, respectively [150].

Th17 cells are the major source of IL-17A (commonly referred to as IL-17) and IL-17F, although other cells, including NK cells and macrophages, were also shown to express IL-17. The IL-17 family of cytokines includes several compounds involved in the protection of mucosal surfaces against extracellular pathogens. There are six known IL-17 family members currently, which are marked with letters from A to F [155]. IL-17A and IL-17F have been implicated in a broad spectrum of inflammatory and autoimmune disease—after linking with their receptors—IL-17RA and IL-17RC, both cytokines can induce secretion of pro-inflammatory cytokines, like IL-6, IL-1, IL-8, TNF-α, and chemokine CXCL1, favoring tissue inflammation, the recruitment of neutrophils, activation of innate immune cells and enhancing B cell functions [156]. In addition, IL-17 signaling induces the release of other inflammatory mediators, like intercellular adhesion molecule 1 (ICAM-1), prostaglandin E2, and matrix metalloproteinases, which may initiate several positive-feedback loops that further increase IL-17 secretion, causing chronic inflammation and tissue damage [157]. Besides IL-17, Th17 cells can also secrete IL-21, IL-22, IL-25, and IL-26 (in humans); however, the majority of pathogenic functions of Th17 cells have been related to the secretion of IL-17 [158]. Because of the important role of IL-17A and IL-17F in inducing tissue inflammation, Th17 cells have been shown to play a critical role in the etiopathogenesis of many autoimmune diseases, in which Th1 was originally considered as a dominant factor. Th17 function depends on the combinations of cytokines expressed in the local environment, and the regulation of these cells differentiation is mediated by a complex cytokine and transcription factor, which may result in both pathologic and protective functions of these cells in inflammatory and autoimmune diseases.

It has been demonstrated that Th17 cells can produce the anti-inflammatory cytokine IL-10, when they were stimulated with IFN-α or IFN-β [159]. On the contrary, IL-23 was shown to reduce the expression of IL-10 in developing Th17 cells, inducing a proinflammatory Th17 subset that may produce IL-17 [160]. Further, Th17 cells exhibit high plasticity—they can differentiate into other T cell subsets in different settings, for example, mature Th17 cells can be transformed by IL-6 into Th1 cells producing IFN-γ [161].

Tregs play a crucial role in immunity tolerance and the control of autoimmunity [162]. Tregs express the signature transcription factor—Foxp3—which is important in their development, differentiation, and regulatory functions [163]. Foxp3 expressing Treg subsets include both naturally occurring Tregs (nTregs) generated in the thymus and induced via post-thymic maturation Tregs (iTregs), which can further differentiate into Foxp3^+^ cells (Th3) and Foxp3^−^ cells, called also Tr1 [164]. Th3 differentiation occurs mainly after oral ingestion of exogenous antigens, and these cells help the secretion of IgA by releasing TGF-β and show suppressive properties in relation to Th1 and Th2 cells [165]. Tr1 cells, being a dominant source of IL-10 in the immune system, play an important role in the inhibition of autoimmunity and inflammation [166]. The immunosuppressive effects of IL-10 are mediated through its impact on downregulation of the expression of MHC-II and co-stimulatory molecules: CD80, CD86, and CD28 on APCs, and the mitigation of activated mast cells, macrophages as well as reduction of the release of their proinflammatory cytokines [167].

TGF-β is produced by nTreg and Th3 cells; however, many immune and non-immune cells may also synthesize this cytokine. TGF-β is needed for the generation of iTregs because the induction of Foxp3 expression driven by TGF-β converts naive T cells into iTregs. This positive feedback between TGF-β and Foxp3 plays an essential role in maintaining peripheral tolerance and maintenance of Tregs [168]. In vivo, TGF-β producing Tregs have been shown to suppress autoimmune T cell responses, inhibit IL-17 production, and enhance the expression of Foxp3 in Th cells [169].

Nowadays, Tregs are recognized as important immunoregulators in many inflammatory and autoimmune diseases, and cellular therapies using these cells are currently undergoing clinical trials for treating these pathologies [170,171]. However, it is worth remembering that some of the cytokines produced by Tregs, including IL-10 and TGF-β, may not always have anti-inflammatory potential, and under certain conditions, they can enhance the function and activity of pathogenic cells. It has been shown that IL-10 can activate B cells, increasing their function as APCs and driving the maturation of B cells into plasma cells [172]. TGF-β is also associated with a number of proinflammatory effects, like the development of IL-17-producing Th17 cells, which promote inflammation [158]. TGF-β can generate IL-9 producing Th cells, which promote tissue pathology. Both TGF-β and IL-10 enhance the survival of CD8^+^ T cells and increase their production of IL-17 and IFN-γ [173,174]. This phenomenon seems to probably be a mechanism by which the immune system maintains its balance.

#### 3.5.2. IDO1, Kynurenines and T Cells

**Figure 3:**
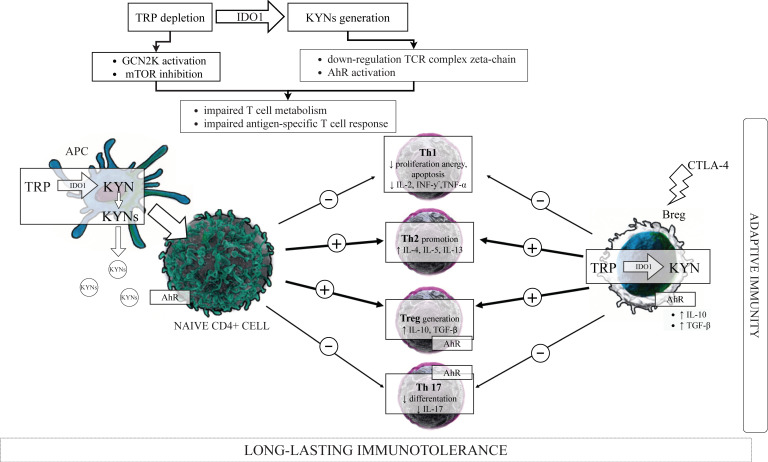
The role of the indoleamine 2,3-dioxygenase-1 (IDO1) and kynurenine pathway metabolites in adaptive immunity. The induction of IDO1 expression on professional APC leads to TRP depletion and KYNs generation in the local microenvironment. Together, TRP deprivation and KYNs-dependent activation of AhR signaling result in metabolic stress sensed by GCN2K, mTOR, and TCR complex zeta-chain, leading to impaired T cells metabolism and their antigen-specific response. The activation of IDO1-dependent KP in APC can directly affect the differentiation of naїve CD4+ T cells by cell cycle arrest and apoptosis of Th1 cells, inhibition of differentiation of Th17 cells while promoting Th2 cells polarization. KYN metabolites, by signaling via the AhR, can also direct the conversion of naїve CD4+ T cells to the immunosuppressive phenotype of Treg and prevent its reprogramming to effector Th17 cell. Similar effects on cells of the adaptive immune system are exerted by IDO1 activation in the Breg, which regulates T cell responses via the release of IL-10 and TGF-β, the suppression of Th1, Th17 cells, and by converting CD4+T cell into Treg. In summary, the IDO1-dependent pathway of TRP degradation may alter Th1/Th2 and Th17/Treg balance and create the local milieu, that is dominated by anti-inflammatory cytokines (IL-10, IL-4, TGF- β), contributing by this way to the long-lasting immunotolerance. Abbreviations: TRP—tryptophan; IDO1—indoleamine 2,3-dioxygenase 1; KYN—kynurenine; KYNs—kynurenines; GCN2K—general control nondepressible 2 kinase; mTOR—mammalian target of rapamycin; APC—antygen presenting cel; Th1—T helper type 1 cell; IL—interleukin; IFN-γ—interferon γ; TNF-α—tumor necrosis factor α; Th2—T helper type 2 cell; Treg—T regulatory cell; TGF-β—transforming growth factor β; AhR—aryl hydrocarbon receptor; Th17—T helper type 17; CTLA-4—cytotoxic T-lymphocyte antigen 4; Breg—B regulatory cell.

As has been above presented, IDO1 induction in cells belonging to the innate immunity led to the depletion of TRP and the generation of KYN and its metabolites (Figure 2), which are the important regulators of adaptive immunity [25], contributing to the long-lasting immunotolerance by several distinct mechanisms (Figure 3).

##### IDO1, Kynurenines, and T Cells Metabolism

One of the earlier theories postulates that TRP breakdown suppresses T cell proliferation by a substantial reduction of the resource of this amino acid in local tissue microenvironments. It has been postulated that TRP-deficient T cells cannot synthesize sufficient proteins for proliferation after antigen presentation by APCs [175]. IDO1-dependent TRP depletion activates the amino acid sensor—GCN2K in CD4^+^ T cells [176]—which controls transcriptional and translational programs coupling cell growth to amino acid availability [177]. Through GCN2K activation, IDO1 can downregulate enzymes participating in fatty acid synthesis in CD4^+^ T cells [176]. Fatty acid synthesis is up-regulated upon T cell activation and is necessary for preventing the death of proliferating cells [178]. Thus, IDO1-dependent activation of GCN2K and reduction of fatty acid synthesis impairs CD4^+^ T cells proliferation and differentiation into effector cell lineages. Fallarino et al. [81] proved that both TRP depletion and the mixture of major TRP metabolites: KYN, 3-HKYN, and 3-HAA are able to induce the GCN2K- dependent down-regulation of T cell receptor (TCR) complex zeta-chain in CD8^+^ T cells, which resulted in impaired cytotoxic effector function of these cells. While CD4^+^CD25^-^ T cells in these conditions were converted to a Treg phenotype through a process requiring GCN2K, a decrease in IL-2 production, and an increase of IL-10 and TGF-β. TRP starvation via IDO1 does not solely act via TCR inactivation, but, in conjunction with induction of Fas, mediates cell cycle arrest in the mid G1 phase leading to T cell apoptosis, clonal anergy, and inhibition of antigen-specific T cell responses [56]. The newer study of Eleftheriadis et al. [179] showed that IDO1, through GCN2K activation, downregulates the levels of TCR complex zeta chain and cMyc, resulting in the reduction of the key enzymes involving in aerobic glycolysis and glutaminolysis, which are required for the rapidly proliferating, activated T cells. The indicated study used a KYN free, APCs-free system of isolated and activated T cells, and authors demonstrated that the direct activation of the GCN2K by TRP is sufficient for inhibition of T cells proliferation, and that this may be an intrinsic cell mechanism for controlling proliferation. Moreover, 3-HAA has been shown to cause immune suppression by inducing apoptosis in T cells through glutathione depletion [80]. Hayashi et al. [180] identified another potential mechanism of 3-HAA action, involving inhibition of 3-phosphoinositide-dependent protein kinase signaling in T cells, which resulted in T cell apoptosis.

In the majority of referred to studies, the immunosuppressive properties of IDO1 were evaluated in a culture media free of fatty acids. However, when free fatty acids were added in cell cultures, IDO1 increased free fatty acid oxidation and although it promoted Tregs differentiation, it did not induce apoptosis or inhibited proliferation of CD4^+^ T cells [181]. Even though IDO1 decreases glycolysis and glutaminolysis by activating GCN2K, it may increase free fatty acid oxidation by activating AhR, providing the necessary energy for CD4^+^ T cell survival and proliferation [182]. Thus, contrary to the previous hypothesis that IDO1-mediated pathways suppressed CD4^+^ T cell function by inducing apoptosis, inhibiting proliferation, and promoting differentiation towards a regulatory T cell phenotype, the more recent data revealed that in a normal environment that contains fatty acids, the immunosuppressive effect of IDO1 cannot be attributed to a decrease in CD4^+^ T cells proliferation and survival.

##### IDO1, Kynurenines, and Th1/Th2 Cells Balance

Experimental data have shown that IDO1 has important immunosuppressive properties involved in immune tolerance and Th1/Th2 regulation. The expression of IDO1 in DCs caused suppression of human T-cell proliferation, creating local immune privilege [75]. IDO1 activity in pDCs blocks the expansion of naive CD4^+^ and CD8^+^ T cells, and the generation of cytotoxic T lymphocytes (CTLs) and Th1 cells, while having less impact on Th2 cells [80]. A similar mechanism was observed concerning IDO1-expressing human eosinophils, which preferentially inhibited Th1 cells but promoted Th2 cells [62]. Moreover, a decrease of Th1 cytokine production and an increase in Th2 cytokine levels has been shown in murine spleen cells after pharmacological inhibition of IDO1 [183]. These results suggested that preferential induction of apoptosis in Th1 cells, but not in Th2 cells, was due to increased susceptibility of Th1 cells to IDO1-induced KYN production or the formation of downstream metabolites of KP [184]. However, in vivo studies on ovalbumin-induced asthma in mice provided contradictory results. IDO1- deficient animals showed weaker Th2 responses in comparison to controls, when challengers with inhaled antigen and their serum levels of antigen-specific IgE were lower, indicating that IDO1-deficiency protected against ovalbumin-induced asthma [185]. While, in another murine model of asthma using the same sensitization, induction of IDO1 expression inhibited Th2-induced asthma [186]. The comprehensive explanation for these contradictory effects was done by MacKenzie et al. [187], who found that during antigens and pathogens presentation by DCs for T cells, naive Th cells are transforming to Th1 subsets, and INF-γ production creates a Th1 dominant microenvironment, inhibiting Th2 differentiation. As IFN-γ induces DCs to express IDO1, a reduction in TRP level, associated with an increase in kynurenines, causes Th1 cells apoptosis and selected survival of Th2 cells, acting as a regulatory loop to limit overactive Th1 cells responses.

Recent evidence suggests that the immunomodulatory properties of IDO1 are largely due to the accumulation of KYN metabolites in conjunction with TRP depletion [32]. It has been shown that KP catabolites are important biological mediators in regulating Th1 and Th2 cell function, although Th2 cells are less sensitive to TRP metabolites [188]. The addition of exogenous KYN metabolites KYN, 3-HAA, QA, 3-HKYN, and PA to T cells cultures showed that compounds could inhibit proliferation and induce apoptosis of active T cells at more physiologically relevant TRP levels than the previous “TRP depletion” theory would suggest [59,110,183,189]. HAA and QUIN induced selective apoptosis in vitro of murine Th1 but not Th2 cells. This process was observed at relatively low concentrations of these kynurenines, did not require Fas/Fas ligand interactions, and was associated with the activation of caspase-8 and the release of cytochrome c from mitochondria [80]. Orihara et al. [190] demonstrated that QUIN was able to reduce Th1 cytokines production, Ca^2+^ flux, proliferation, and survival of Th1-like cells through increased induction of cell death, whereas Th2-like cells were spared, leading to increased Th2-like dominance. Taking together, the shift of Th1/Th2 balance favoring Th2 cells survival evoked by KP activation seems to limit the uncontrolled activation of adaptive immunity.

It should be emphasized that described effects of KP metabolites on function and viability of cells of the adaptive immune system can be partly mediated by AhR, which are expressed in certain subtypes of T cells, such as naive Th, Th17, and Treg cells, whereas fully differentiated Th1 cells fail to up-regulate AhR after activation and cannot be directly modulated by AhR ligation [191]. The activated AhR suppresses immune responses under normal conditions, whereas reduction of AhR activity enhances these responses [192]. However, the results of the studies investigating the role of AhR in modulating the immune response are sometimes divergent. Activation of the AhR by environmental toxins differs from that seen following stimulation with its natural ligands, for instance, AhR activation of T cells by dioxin was shown to inhibit immunity by the generation of Tregs, whereas it worsened immunity following activation by 6-formylindolo [3,2-b]carbazole (FICZ), an endogenous ligand derived from TRP [193]. In agreement with this theory, Ambrosio et al. [194] found that dioxin treatment of *Trypanosoma cruzi* infection in mice resulted in the increased death of activated T cells and elevated number of Tregs producing TGF-β. The weak AhR ligand—3-HKYN—was also able to induce Tregs and improve the unbalanced ratio between activated T cells and Tregs during the chronic phase of the infection, but it is only partially efficient in controlling the parasitemia and is unable to eradicate it. Moreover, a negative effect of a strong AhR activation on the development of memory CD8^+^ T cells was also observed. AhR ligation restricted the differentiation of CD8^+^ memory T cells, probably by indirect, AhR-dependent regulation of DCs, similar to this observed with Th1 cells [193].

##### IDO1, Kynurenines and Tregs/Th17 Cells Balance

IDO1 contributes to immune regulation by assisting Tregs effector function. In murine pDCs treated with TGF-β, IDO1 can create signaling for long-term immune tolerance by transforming CD4^+^ T cells into immunosuppressive Foxp3^+^ Tregs [81,195], which, in turn, are able to induce IDO1 expression in pDCs and neutrophils [83]. Functionally inactive Tregs acquired potent suppressor activity when cocultured with IDO1-expressing pDCs. It is worth noting that IDO1-competent pDCs prevent effector T cells response and promote Tregs differentiation only when local conditions or treatments induce pDCs to express IDO1 and that GCN2K signaling were also pivotal for Tregs activation. Moreover, this IDO1/GCN2K-dependent process of Tregs activation was MHC-restricted and was prevented by CTLA4 blockade [196]. The B7 receptors on IDO1-positive DCs bind to CTLA4 on Tregs causing them to proliferate, and the blockade of the CTLA4/B7 axis had a negative impact on IDO1 enzymatic activity and Tregs activation, indicating that CTLA4^+^ Tregs ligate B7 on pDCs to maintain IDO1 activity in pDCs [84]. Tregs activated by IDO1 remarkably upregulated PD-L1 and PD-L2 expression on target DCs, and the ability of Tregs to suppress T cells proliferation was abrogated by antibodies against the PD-1/PD-L pathway but was not dependent on IL-2, IL-10, or TGF-β [196]. Therefore, IDO1 activity in pDCs promotes de novo Treg differentiation from naive CD4^+^ precursors, and the same results occurred when naive CD4^+^ precursors were cultured with low TRP/high kynurenines medium, directly implicating TRP catabolism in Tregs generation [81]. Moreover, IDO1 expression was shown to block the conversion of Tregs to Th17 cells by activation of the GCN2K pathway and suppression of IL-6 production in pDCs [82]. In this manner, IDO1 does not only suppress effector T cells directly but also indirectly may influence Tregs suppressor activity concerning Th1, Th2, or Th17 cells. However, the inhibition of T cell response/proliferation seems to be dependent upon the microenvironment, since the exposure of Tregs to proinflammatory IL-6 is recognized to switch mature Tregs into a phenotype recalling Th17 cells [197]. In turn, KYN resulting from the activation of IDO1 promoted per se IDO1 expression through an agonistic action on AhR in DCs [77,78,198], creating a positive loop reinforced IDO-mediated effects in these cells. Ligand activation of AhR both on T cells and pDCs have been reported to contribute to Tregs development and Th17 suppression [199,200]; however, it has also been demonstrated to activate IDO1 in DCs [198], suggesting a forward loop in KYN-induced AhR activation. In line with these, the protective role of IDO1 activation in experimental autoimmune encephalomyelitis (EAE) in rats has been demonstrated [201], and IDO1 expression in DCs induced by estrogen administration led to concomitant T cell apoptosis associated with EAE suppression and decreased rate of relapses during pregnancy [202]. In contrast, the pharmacological blockage of IDO1 led to increased Th1 and Th17 responses, decreased Treg responses, and EAE exacerbation overall [203].

KYNA has also been identified as a potent agonist of the AhR [148], nevertheless, studies directly demonstrating the possible AhR-mediated effect of KYNA on the modulation of the Treg/Th17 axis were lacking. While, KYNA has been reported to decrease IL-17 expression in activated T cells and to deplete Th17 cells in another way—by acting on G-protein-coupled receptor 25 (GPR35) on DCs, causing the suppression of their IL-23 production [204]. Regardless, the recent study of Engin et al. [205] showed that the accumulation of KYNA, due to overexpression of the IDO1 by AhR activation, induces the AhR/IL-6/STAT3 signaling pathway and differentiation of naive CD4+ T cells toward Th17 cells. Whereas it inhibits Tregs, leading to Treg/Th17 imbalance and cytokine storm, which causes the fatal consequences in SARS-CoV-2 infection. This new finding suggests that KYNA may play an opposite role to KYN in modulating the balance of the Treg/Th17 axis. This is in line with the previous observation that IDO1 plays a vital role in the conversion of Tregs into Th17 cells by blocking IL-6 production, which is needed for this conversion. The phenotype of reprogrammed Tregs after IDO1-blocking have been described as resembling “multifunctional T-helper cells”, co-expressing different cytokines, like IL-2, IL-17, IL-22, and TNF-α [206].

Another downstream KYN metabolite—3-HAA—has been shown to diminish Th1 and Th17 responses and elevate the Treg response, in part by the indirect action of DCs. The administration of this compound resulted in an amelioration of EAE in mice [203]. DCs treated with 3-HAA in vitro reduced their IL-6 production and increased expression of TGF-β. Moreover, when 3-HAA-treated DCs were cocultured with naive CD4^+^ T cells, the generation of Tregs was stimulated [203]. These results demonstrated that IDO1, by the generation of 3-HAA, can enhance TGF-β expression in DCs and promote Tregs differentiation. Moreover, the therapy with N-(3,4,-dimethoxycinnamoyl) anthranilic acid, an orally active derivative of 3-HAA analog (tranilast), likewise demonstrated a suppressive effect in EAE, with fewer and milder relapses observed in the treated animals [207].

Similarly, cinnabarinic acid, a less known endogenous KYN metabolite, was capable of protecting against EAE by enhancing Tregs at the expense of Th17 [208].

In summary, both KYN and its downstream metabolites affect the balance of the Th17/Tregs system, shifting this balance in favor of the immunosuppressive Tregs.

### 3.6. Kynurenines and IL-2 Signaling

The memory CD4^+^T cells are critical to ensure long-lasting immune protection, and their depletion is linked with persistent inflammation. The survival of the memory CD4^+^ T cells depends on signals provided by the γ-chain-receptor cytokines, such as IL-2 [209]. Dagenais-Lussier and coworkers [210] showed that the increased production of KYN correlates with defective IL-2 signaling in memory CD4^+^T cells from HIV-infected subjects, leading to their Fas-mediated apoptosis. The treatment of memory CD4^+^T cells with the physiological concentration of KYN (5 μM) in vitro inhibited IL-2 signaling through the mechanism related to the production of ROS [210].

Altogether, presented herein data indicate that IDO1 activation can transform the function of APCs and convert local T cells’ function from an immunogenic one to a tolerogenic one. However, KP enzymes downstream of IDO1 can also initiate tolerogenesis by DCs independently of TRP deprivation. The paracrine production of kynurenines might be one mechanism used by IDO1-competent cells to convert DCs lacking this functional enzyme to a tolerogenic phenotype within an IFN-γ-rich environment [211]. On the other hand, some studies identified IDO1-specific CD4^+^ and CD8^+^ T cells in both healthy people and cancer patients that are capable of removing IDO1-expressing cells, including IDO1-positive DCs and tumor cells. This anti-IDO1 immune response probably represents a counter-regulatory mechanism, aimed at limiting IDO1-mediated immune suppression in order to reinforce the antigen-specific immune response [212,213,214].

### 3.7. IDO1 and B Cells

While the majority of the literature has focused on investigating the suppressive effects of IDO1 related to T cells, several studies are evaluating the role of IDO1 in B cells’ response. The primary function of B cells in the production of antibodies. Notwithstanding, a subpopulation of B cells that regulate immune responses independently of antibody production has been identified [215]. These cells, termed regulatory B lymphocytes (Bregs) were discovered based on their ability to inhibit effector immune processes [216] through IL-10-based mechanism, which is responsible for down-regulation of inflammation [217]. Beyond the IL-10 production, there were some suggestions that part of this immunosuppressive effect of Bregs is dependent on interactions with other regulatory cell lineages; they may suppress Th1 and Th17 differentiation and exert the direct inhibitory effect on antigen presentation by DCs, whereas they induce Tregs differentiation [218].

In 2009, Scott et al. [219] observed that pharmacological inhibition of IDO1 activity had the unexpected consequence of ameliorating arthritis symptoms in the rheumatoid arthritis model in mice. This reduction of arthritis symptoms was resulted from a diminished autoreactive B cell response, reflecting as decreased autoantibody titers, whereas no difference was detected in the percentage of Tregs, nor in the levels of Th1/Th2/Th17 cytokines. In contrast, cytokines associated with inflammation, like MCP-1, IL-6, and IL-10, were reduced in these mice. This study demonstrated that IDO1 plays an activating role in establishing the autoreactive B cell profile at the onset of the autoimmune response, indicating its previously unappreciated role in the stimulation of B cell function. This finding suggested that IDO1 is not simply immunosuppressive but rather plays a more complex role in modulating inflammatory responses, especially driven by autoreactive B cells.

A year later, Vinay et al. [220] demonstrated the existence of a murine B lymphocyte subpopulation, in which IDO1/IDO2 is induced at the mRNA level upon stimulation with CTLA-4 immunoglobulin, but neither protein expression nor enzymatic activity was evaluated in this study. CTLA-4 is a central inhibitory regulator of T cell proliferation and expansion, and the CTLA-4 pathway through ligation to CD80 and CD86 on APCs can upregulate Foxp3 expression induced by TGF-β, leading to induction of Tregs [221]. Additionally, CTLA-4 engagement of B7 ligands on DCs, through the induction of the IDO1, may involve the maintenance of peripheral tolerance [83]. Godin-Ethier and coworkers [222] confirmed that both IDO1/IDO2 genes and IDO protein can be up-regulated in human B lymphocytes in response to T cell signals; however, they reported only weak/absent enzymatic activity from these IDO-expressing cells, concluding that IDO may not be a counter-regulatory mechanism used by B lymphocytes to down-regulate immune response.

In contrast to Godin-Ethier et al. [222], Nouël and coworkers [65] reveal a novel regulatory pathway in B cells, mediated by the TGF-β/IDO1 axis in a CTLA-4 dependent manner. They showed for the first time that CTLA-4 induced B-cells can produce IDO1 and become effective induced regulatory B cells (iBregs), which were able to generate Tregs, Tr1, and Th3 cells when were cocultured with T cells, whereas they suppress the induction of Th1 cells. These authors also showed that the TGFβ/IDO1 axis plays important role in mediating durable regulatory functions in B cells, indicating new perspectives for future management of autoimmune diseases [65]. It has been also noticed that IL-21 may induce a Breg phenotype in human B cells, which is associated with the expression of immunoregulatory molecules: granzyme B, IL-10, and IDO1, and that the granzyme B-dependent degradation of the TCR complex zeta-chain may suppress T-cells proliferation [223]. Similarly, the mesenchymal stromal cells can promote the survival and proliferation of Bregs, and IDO1 partially participates in this effect [224]. Piper et al. [225] identified AhR as a relevant contributor to the transcriptional regulation of differentiation and function of IL-10- producing Bregs. They showed that mice with AhR deficiency in Bregs develop exacerbated arthritis, associated with significant reductions in IL-10-producing Bregs as well Tregs, and show an increase in Th1 and Th17 cell subsets compared with mice, which have AhR-sufficient Bregs.

The recent in vivo studies performed on the models of autoimmunity suggest that IDO2 may play a distinct from IDO1 role in the B cell-mediated autoimmunity. It has been shown that IDO2 may be a proinflammatory molecule contributing to autoreactive B cell responses. This pathogenic function of IDO2 was described by Merlo and colleagues in the KRN model of autoimmune arthritis [226] and collagen-induced arthritis [227]. IDO2 knockout mice display decreased joint inflammation, reduction the autoreactive B cells, and lower pathogenic autoantibodies levels compared to wild-type mice, indicating pathogenic IDO2 function in autoantibody-mediated autoimmunity [226]. The administration of IDO2-specific autoantibodies alleviated arthritis in two independent preclinical arthritis models, reducing autoreactive T and B cells activation [227]. In the same way, the anti-IDO2 3DNA formulation ameliorates arthritis in a preclinical model [228]. The recent study of this team using double IDO1/IDO2 knockout mice revealed contrasting roles of IDO1 and IDO2 in immunity: IDO1 mediates T cell suppressive effects (probably by KYN production), whereas IDO2, which practically does not produce KYN, works directly in B cells as a proinflammatory mediator of autoimmune processes. Thus, IDO2 seems to be the dominant player in the pathogenic autoantibody-mediated autoimmunity through an IDO1-independent mechanism [229].

## 4. The Role of IDO1 and KP Activation in Autoimmunological Endocrinopathies

### 4.1. T1DM—An Autoimmune Disease with Unclear Pathophysiology

T1DM is an autoimmune disorder, which results from the breakdown of immune tolerance that leads to the selective destruction of β-cells in the pancreas and disturbances in insulin secretion with consequent severe impairment of glycemic control. In the asymptomatic preclinical phase, the influx of immune cells to the pancreatic islets of Langerhans takes place, and this process precedes hyperglycemia and disease onset. However, the circumstances driving this immune alteration are still poorly explained [3,9,230].

The classical hypothesis for the development of T1DM was that in individuals with the genetic predisposition, the activation of the immune system (T-cells mediated autoimmune disease) by one or multiple environmental triggers results in the destruction of the pancreatic β-cells [231]. The discovery of pancreatic islet cell autoantibodies directed against different autoantigens [11] constituted a strong argument that β-cells -specific proteins and peptides were targeted by the immune system [232]. In agreement with this hypothesis, the peripheral immune regulation appears defective in T1DM patients, and the disturbing crosstalk between cells of adaptive and innate immunity may accelerate or delay T1DM development [24]. However, immuno-based therapies in subjects at high risk of developing T1DM delay the progression to the overt disease but not prevent the onset T1DM [233].

The data from recent studies pointed out on the role of β-cells as a key contributor to the T1DM. Abnormal pancreatic β-cells may influence the normal function of the immune system in such a way, that it will need to clear these dysfunctional cells. Several recently performed studies seem to support this theory, for example, the smaller pancreatic volumes in persons at risk of T1DM [234]. The induction of endoplasmic reticulum stress has been recognized as a major contributory factor to β-cells dysfunction in the early stage of T1DM [235], and resulted in formulation an alternative “β-cells centric hypothesis” [236]. According to this theory, once the β-cell is under attack, an inflammatory environment is formed that appears to favor the release of additional proinflammatory cytokines and chemokines by the β-cells, attracting more immune cells. In the inflammatory state, β-cells present higher exposure of human leukocyte antigen (HLA) class I molecules, creating additional signaling for residual cytotoxic CD8^+^ T cells, whose frequency are increased in the pancreata of patients with T1DM compared with those of healthy controls [237]. Tregs, which have an important role in repressing these autoreactive T cells in healthy conditions, show a reduced suppressive capacity in patients with T1DM [238], suggesting that insufficient immune regulation can be the reason for an intensified autoimmune response exerted by autoreactive T cells. This theory is supported by the fact that patients with cancers, treated with immune checkpoint inhibitors for enhanced immune response and reduced immunosuppression, are at risk of developing T1DM due to loss of immune regulation combined with activation of an immune response against the tumor tissue [239]. The more recent study of Li et al. [240] found that β-cells can actively participate in T1DM development. Under stressed conditions, β-cells produce neoantigens and are able to upregulate the expression of MHC I/II and co-stimulatory molecules that are normally exhibited by the professional APCs. This subset of APC-like β-cells works together with pDCs at the cellular level to activate CD4^+^ and CD8^+^ T cells, initiating early autoimmune responses leading to T1DM development. This view, being in accordance with theory of Roep et al. [236], revisited the classical hypothesis of the T1DM development that assumed that β-cells are only a passive participant during T1DM onset.

The combination of these both theories was postulated by Peters et al. [241], who believe that T1DM is probably the result of a complex network of dysfunctions both in the β-cells and the immune system, with defects in both innate and adaptive immunity.

### 4.2. IDO1 and T1DM

Although an impaired IDO1-mediated TRP metabolism has been observed in distinct autoimmune diseases [28], so far there are not much data in the available literature, concerning the role of IDO1 and the activation of KP in autoimmunological endocrinopathies. Among the known endocrinopathies, T1DM is an autoimmune disorder, in which the significance of IDO1 activation is relatively well described. In general, IDO1 is recognized as a regulator of immunity—it not only produces immunoregulatory kynurenines, but it also acts as a signal-transducing molecule, promoting immunotolerance in pathophysiological conditions [242,243]. Nevertheless, the inflammatory state that characterizes the preclinical phase of T1DM can affect IDO1 protein expression and activity, impairing its role in immune tolerance in the pancreas.

The preclinical studies in the field of T1DM are carried out in different experimental settings using models of nonobese diabetic (NOD) mice. The model has been described as a prototypic model of autoimmune diabetes, which resembles the T1DM course in humans [244]. A large proportion of female mice generally dies of type 1 diabetes, reflecting the onset of severe insulitis about 4 weeks of age, which is associated with T cells-mediated destruction of pancreatic β- cells. The predisposition of NOD mice to develop autoimmunity is the result of defects in both peripheral and central tolerance mechanisms [245]. Several abnormalities have been described in those animals, like abnormal APCs function [246], lymphocyte accumulations around the islets of Langerhans [247], or generation and function of Tregs in the periphery [248]. Data obtained from this spontaneous model of diabetes clearly indicate that monocytes, macrophages, and pDC play a key role in the development of this disease [249].

Using NOD mice during the prediabetes phase, Grohmann et al. [250,251] observed that IFN-γ fails to induce tolerizing properties in their DCs. This effect was associated with low IDO1 activity and impaired TRP catabolism by transient blockade of the STAT1 pathway of intracellular signaling by IFN-γ, caused by peroxynitrite production. The use of a peroxynitrite inhibitor restored both suitable TRP catabolism and tolerance in those mice. There were the first reports of experimental diabetes, linked defective immunotolerance to impaired TRP catabolism. A similar observation was done by Fallarino and coworkers [252], who used CTLA-4, another IDO1 inducer. Subsequently, Hosseini-Tabatabaei et al. [253] clarified this phenomenon, showing that defective TRP metabolism can be attributed to the impaired ability of IFN-γ to induce IDO1 expression in both DCs and fibroblasts of these animals by a mechanism related to defective STAT1 phosphorylation in the IDO1 signaling pathway. The protective role of IDO1 in the development of autoimmune diabetes was also confirmed in a streptozocin-induced model of diabetes. Fallarino et al. [254] identified IDO1 as the critical Toll-like receptor 9 (TLR9) downstream effector in regulating autoimmunity. In diabetic animals, the disease progression was accompanied by up-regulation of IDO1 in pancreatic lymph nodes, and it has been exacerbated by in vivo administration of an IDO1 inhibitor. Conversely, signaling through TLR9 induces IDO1 expression in splenic DCs and attenuated the disease in an IDO1-dependent fashion. However, TLR9-deficient mice developed a severe form of the disease, accompanied by a lack of IDO1 induction in pancreatic lymph nodes [254].

The maneuvers capable of the preservation of adequate levels of the IDO1 in NOD mice have been shown to restore autoantigen-specific tolerogenesis by DCs in vivo. Pallotta et al. [255] demonstrated that up-regulation of IDO1 expression and enzymatic function in pDC of NOD mice may restore their function, resulted in decreased production of proinflammatory cytokines and suppression of the presentation of β-cell autoantigens in vivo. The administration of a proteasome inhibitor—bortezomib—to prediabetic NOD mice caused the prevention of diabetes onset through a mechanism related to restoration of IDO1 expression in pDCs from these animals and reinstallation immune tolerance to pancreatic autoantigen [256]. In the same way, the use of dermal fibroblasts with stable IDO1 expression as a cell therapy in NOD mice by Zhang et al. [257] resulted in the elevation of plasma KYN levels and had a protective influence on islet β-cells, which has been guarded against toxicity induced by both autoreactive T cells and the proinflammatory cytokines. Additionally, they successfully inhibited CD8^+^ T cells, Th17 cells as well as increased Tregs in different organs of NOD mice. The injections with a higher dose of IDO1-expressing fibroblasts were able to restore normoglycemia in a high percentage of NOD mice. Moreover, the transplantation of IDO1-expressing islets can prolong the islet graft survival, and this protection is attributed to the local modulation of TRP catabolism [258,259]. Fallarino et al. [260] implanted peritoneally Sertoli cells, which provide local immunological protection into NOD mice, and observed the prevention and reversion of diabetes and the normalization of glycemia in these animals. This effect was associated with restoration of systemic immune tolerance, and it was dependent on efficient TRP metabolism in the xenografts, increased TGF-β secretion followed by autoantigen-specific Tregs differentiation, and recovery of β-cells function in the diabetic recipients. The administration of human chorionic gonadotropin, a key pregnancy hormone to NOD mice inhibited the activation of diabetogenic CD4^+^ and CD8^+^ T-cells in vitro, and the progression of T1DM in vivo by upregulating the expression of IDO1 in DCs [261]. In the recent study, Lemos et al. [262] used DNA nanoparticles, which activate the signaling adaptor stimulator of interferon genes (STING) and demonstrated that such treatments elevated IDO1 activity, which regulated T cells immunity in spleen, pancreas, and pancreatic lymph nodes of NOD mice. Moreover, this treatment delayed T1DM onset and reduced T1D incidence when administered before disease onset. This study also revealed that NOD mice possess STING polymorphism that may be partly responsible for insufficient interferon expression and IDO1 induction.

On the other hand, emerging evidence supports that β-cells destruction caused by autoimmune responses can be rectified by AhR signaling. In the recent comprehensive review, Yue et al. [263] described the potential implication of AhR activation in T1DM pathogenesis, presenting its regulatory mechanisms in different types of immune cells. AhR activation by its ligands not only modulates the development and functionality of immunosuppressive cells, but also reduces the expression of pro-inflammatory cytokines, and by this way attenuates autoimmune responses during the course of T1DM development. However, the T1DM-prone NOD mice show the reduced activity of AhR [264], which creates the need to search for new, safe compounds that could activate AhR and fight the autoimmune responses.

In summary, all these results suggest that in T1DM-prone NOD mice the insufficiency in IFN-γ/IDO1/AhR axis is present, thus any attempts to the reinforcement of this axis in appropriate cells of the immune system could be one of the ways of preventing T1DM in this model, through the restoration of the immunotolerance to pancreatic autoantigens.

Effective immunological suppression strategies have been used to protect against T1DM onset. For this purpose, the chimeric vaccines that link immuno-stimulatory molecules with autoantigens to enhance vaccine efficacy were developed. The linkage of cholera toxin B-subunit to the diabetes autoantigen proinsulin generated a fusion protein, which was able to protect against T1DM [265,266,267]. The oral immunization with this vaccine effectively suppressed β-cell destruction and clinical diabetes in adult NOD mice [265,267]. Additionally, vaccine-induced IDO1 expression in DCs was associated with the induction of immunological tolerance [266,268]. Comparable results were obtained by the team of Ghazarian et al. [269] who showed that the activation of invariant natural killer T (iNKT) cells at the time of infection caused by pancreatic enterovirus—Coxsackievirus B4—in a subset of proinsulin 2-deficient NOD mice can prevent diabetes development. They observed that during diabetes onset in these mice, the infiltration of pancreatic islets by inflammatory macrophages, producing high levels of pro-inflammatory cytokines (IL-6, IL-1β, TNF-α) has occurred, which was associated with the activation of T cells producing anti-islet autoantibodies. Although the viral infection itself accelerated the development of diabetes, the presence of stimulated iNKT cells during this time caused infiltrated macrophages to express several suppressive enzymes, among which IDO1 was sufficient to inhibit anti-islet T cells response and to prevent T1DM. This study suggests that IFN-γ, the strong activator of IDO1 expression, can play a protective or deleterious role in diabetes development. The strong IFN-γ release early after viral infection upregulates IDO1 expression to downregulate the virus-induced inflammation. However, if at this time iNKT cells are inactive, the production of pro-inflammatory cytokines may increase the recruitment and activation of pathogenic T cells, producing IFN-γ. In these conditions, IDO1 is no longer expressed in the pancreas, and IFN-γ production will lead to β-cells destruction [269].

Another strategy used to counteract the development of T1DM was modulating the gut microbiota. Dolpady et al. [270] administered orally *Lactobacillaceae*-enriched probiotic to NOD mice and showed that the modification of gut microbiota inhibited IL-1β expression, while it enhanced release of IDO1 and IL-33 from the inflammasome. Those modifications of the intestinal microenvironment promoted differentiation of tolerogenic DCs with simultaneous reduction of Th1 and Th17 cells expansion in the intestinal mucosa and within the pancreatic lymph nodes. These results pointed out a new therapeutic possibility the use of probiotics to counter-regulate autoimmunity and prevent T1DM.

Observations made on animal models were confirmed during clinical studies in patients with T1DM. In humans, IDO1 expression and activity are known to exhibit relatively large interindividual variability, often as a result of single nucleotide polymorphisms (SNPs) in the enzyme gene, especially under pathological conditions [271,272]. Orabona et al. [273] discovered that, in children with T1DM, the IDO1 expression and protein levels were very low or absent in peripheral blood mononuclear cells (PBMCs) in response to IFN-γ. The IDO1 defect correlated with a higher IL-6 receptor expression, and children with SNPs in IDO1 are at an increased risk of developing T1DM. In T1DM patients sharing such a common IDO1 haplotype, incubation of PBMCs in vitro with tocilizumab, a humanized antibody that blocks IL-6 receptor, rescued IDO1 activity. In the same study, the treatment of NOD mice with tocilizumab normalized glycemia via IDO1-dependent mechanisms. Thus, the functional SNPs of IDO1 were associated with defective TRP catabolism in human T1DM, and the therapeutic effect of tocilizumab required an intact IDO1 expression. Anquetil et al. [274] also reported a deficient IDO1 expression in human β-cells of T1DM patients as compared to healthy controls. IDO1 expression was mainly present in insulin-producing cells and nearly absent from insulin-deficient islets in human pancreatic tissue, especially in patients with multiple autoantibodies against β- cells. Moreover, a progressive loss of IDO1 expression was observed during the course of T1DM, with a significant decline of IDO1 at a time just preceding β-cells destruction [274]. Zoso et al. [275] described and characterized a population of human MDSCs, named fibrocytic MDSCs, which transcriptionally lie between DCs, macrophages, and fibrocytes. This MDSC subset promotes Tregs differentiation from naive CD4^+^ T cells and induces normoglycemia in a xenogeneic mouse model of T1DM. In order to exert their strong protolerogenic function, fibrocystic MDSCs require direct contact with activated T cells, which leads to the expression and secretion of IDO1.

In monocytes and pDC derived from peripheral blood of T1DM patients, Badal and colleagues [276] observed reduced expression of IDO1, which testified that these cells have diminished tolerogenic capacity as compared to their normal healthy counterparts. In contrast, pDCs of this same T1DM group showed a significantly higher frequency of pDCs expressing IFN-α than healthy controls, whereas the monocytes had a comparable to controls frequency of IFN-α-expressing cells. Interestingly, following in vitro stimulation with self-DNA from dead β-cells and antimicrobial peptide LL37 (DNA-LL37) complexes, both monocytes and pDCs from T1DM patients demonstrated higher IFN-α expression. Furthermore, the poststimulatory ability for antigen presentation and the co-stimulatory ability of these cells was higher in the T1DM group than in controls, and, upon coculture, they were able to activate autologous CD4^+^ T cells and induce apoptosis of cultured β-cells. These results support the undeniable role of a disturbed balance between the cells belonging to the innate immunity system, that may involve both immunotolerance by the expression of IDO1, or can be skewed towards pro-inflammatory phenotype by the expression of IFN-α under certain circumstances.

Taking into consideration all these data from the animal models and human studies, it seems that restoration of IDO1 immunoregulatory mechanisms may be clinically beneficial in patients with T1DM.

### 4.3. IDO1 and Autoimmune Thyroiditis

Hashimoto’s disease and Graves’ disease are the most common and extremely different forms of autoimmune thyroiditis, that lead to thyrocyte death or hyperfunction, respectively [4]. So far, only a few studies exist in which the role of IDO1 in the onset of these diseases was investigated.

In patients with GD, the ratio of serum KYN to TRP, as well as IDO1 expression in B cells and DCs, were increased as compared to healthy subjects. CD4^+^ T cells derived from GD patients have enhanced tryptophanyl-tRNA synthetase (TTS) expression and their proliferation was not inhibited in the presence of IDO1-expressing DCs. In contrast, CD4^+^ T cells derived from healthy controls had low TTS expression, and their proliferation was inhibited under similar conditions [277]. Because TTS can functionally antagonize IDO1-mediated immunosuppression by TRP reservoir formation, the authors concluded that increased TTS expression in CD4^+^ T cells may prevent IDO1-mediated immunosuppression, linking disturbed TRP metabolism to a pathogenic mechanism involved in GD development. However, in another study, the lower KYN to TRP ratio and a significant increase in TRP levels were detected in sera from HT and GD patients as compared to matched controls [278]. The patients, mainly those with severe disease, show a diminished number of peripheral pDCs and a defective expression of several immunoregulatory molecules, including IDO1 by these cells. While more pDCs and a diminished expression of regulatory molecules were detected in thyroid tissue from these patients. These data suggest that the abnormal proportion and phenotype of pDCs may contribute to the pathogenesis of autoimmune thyroiditis.

Interestingly, the symptoms of GD, similarly to other autoimmune diseases, significantly ameliorate during pregnancy and reappear at postpartum, due to the fact that placenta syncytiotrophoblasts can synthesize the immunologically active molecules, including IDO1, which suppress immune responses. In contrast, no clinical change in HT occurs during pregnancy, although the dose of levothyroxine needs to be increased during the pregnancy, similarly as in all forms of hypothyroidism [279].

Coppola et al. [280] evaluate in vitro the ability of human fibroblast-like limbal stem cells, the immune-privileged phenotype, to exert immunomodulation on PBMCs from female HT patients and healthy controls. Following exposure to Th1 cytokines, these cells expressed different cytokines, including IDO1, maintaining their negative phenotype for MHC class II and costimulatory molecules. During coculture, these cells suppressed proliferation in healthy activated PBMCs, whereas the Th imbalance of autoreactive T cells from HT patients was fully restored. These results indicated the inappropriate activation of autoreactive T lymphocytes in inflammatory milieu generated in HT, and suggest that the creation of a tolerogenic environment can reverse disease progression.

The experimental autoimmune thyroiditis (EAT) has been studied using a mouse so-called NOD-H2^h4^ model that develops spontaneously. These animals lost the spontaneous development of diabetes but acquired thyroiditis. Autoimmune thyroiditis in these mice is a T cells-mediated autoimmune disease that results in the destruction of the thyroid follicles [281].

It has been demonstrated that CTLA-4 blockade exacerbated autoimmune thyroiditis in NOD-H2^h4^ mice and induced a strong expression of IDO1 in mouse thyroid glands and peripheral APCs. Moreover, the intensified IDO1 expression was also observed in the thyroid gland from patients with metastatic melanoma, who had received treatment with a CTLA-4 blocking antibody. The authors interpreted this IDO1 increase as a counterregulatory mechanism, protecting against an excessive inflammation induced by the CTLA-4 blockade. Similarly, NOD-H2^h4^ mice developed an attenuated form of thyroiditis when injected with an adenovirus expressing IDO1 directly into the thyroid gland after the beginning of iodine supplementation in the drinking water. The local expression of this immunoregulatory molecule efficiently protects the thyroid glands from autoimmune attacks but does not impact systemic immunity [282]. Recently, Qiu et al. [283] documented the role of IDO1-induced Tregs expansion in *Prunella vulgaris*-mediated attenuation of experimental autoimmune thyroiditis in rats. They showed that administration of this herbal compound induced IDO1 mRNA and protein expression in the spleen and intestine, increased serum KYN/TRP ratio and production of IL-10 and TGF-β, and promoted the expansion of splenic Tregs. Interestingly, IDO1 mRNA levels and KYN/TRP ratio were comparable between healthy controls and non-treated rats with EAT. As explained by the authors, the enhanced IDO1 expression was a compensatory mechanism, by which rats with EAT tried to reduce the self-activated immune response at the beginning of the disease. These counterregulatory mechanisms have been likely exhausted during EAT development, leading to the reduction in IDO1 expression to the level detected in healthy animals.

In the light of the few above studies, it seems that the local IDO1 expression could efficiently protect the thyroid glands from autoimmune attacks. This hypothesis is supported by a study conducted on thyroid carcinomas tissue and thyroid carcinoma cell lines [284]. IDO1 gene expression was higher in the thyroid carcinoma tissue compared with normal thyroid, and it was associated with Foxp3^+^ Tregs density in the tumor microenvironment. IDO1 was also expressed in human thyroid cancer cell lines in vitro, and in a cell line with the highest IDO1 expression, the increased KYN level was also detected in the cell culture medium, indicating functional IDO1 activity. The coculture of this cell line with activated T lymphocytes resulted in the blocking of lymphocytes proliferation, whereas Tregs differentiation was increased. The above-mentioned immunoregulatory effect was mediated by the soluble factor—KYN.

According to our best knowledge, in the available literature there are no data so far concerning the significance of IDO1-mediated KP activation in the onset and progression of other autoimmune endocrinopathies, with exception of study Gupta et al. [285], demonstrating IDO1 reactivity in pancreatic ducts of patients with type 2 autoimmune pancreatitis.

## 5. Conclusions and Future Perspectives

Autoimmune diseases typically result from the loss of self-tolerance, which leads to the generation of self-reactive lymphocytes and the production of autoantibodies that cause tissue damage. IDO1- mediated activation of KP has proven important in the linking innate and adaptive immune processes, such as inhibition of T cell responses to antigenic stimulation, modulation of APC functions, generation and maintenance of Treg suppressor activity, and inhibition of proinflammatory cytokines production. Thus, manipulating IDO1/KYN/AhR axis seems to be a promising strategy to treat a range of chronic autoimmune diseases, including autoimmune endocrinopathies. Although most of the studies demonstrating a relationship between alterations of TRP metabolism via KP and immunoregulation have been carried out in vitro or in experimental animal models, several of the collected data indicate that they can be transferred to humans. This opens interesting possibilities for therapeutic applications of IDO1 inducers in conditions, where immunotolerance mechanisms fail, such as autoimmune endocrinopathies. Alone, or in combination with other already existing therapies, this approach might create a new therapeutic combination, that will involve several aspects of the pathogenic process, providing more complete protection and possible prevention of the disease onset.

## Data Availability

Not applicable.
